# Supplementary material for: An autoregulatory negative feedback loop controls thermomorphogenesis in Arabidopsis
Source: PLoS Genet. 2021 Jun 1;17(6):e1009595. doi: 10.1371/journal.pgen.1009595 (PMC8195427; doi:10.1371/journal.pgen.1009595)
Supplement: S1 Table — (PDF) [file pgen.1009595.s001.pdf]

S1_Table: primers used in this study.

Oligonucleotides	
For qRT-PCR	
ACT7-F TCCATGAAACAACTTACAACTCCATCA	[1]
ACT7-R CATCGTACTCACTCTTTGAAATCCACA	[1]
HEC1-F GAGGAAGGGTTTTGATCGGTGGAG	This study
HEC1-R TGCATTGCCCACCATCTGATGAGT	This study
GFP-F GTGAAGGTGATGCAACATACGG	This study
GFP-R AAGTCGTGCCGCTTCATATGA	This study
HEC2-F GAGGAATGACGGCGGTGGC	This study
HEC2-R TGATCAGACCGCATAATGCCACAC	This study
YUC8-F TGTATGCGGTTGGGTTTACGAGGA	[2]
YUC8-R CCTTGAGCGTTTCGTGGGTTGTTT	[2]
HFR1-F ATTGGCCATTACCACCGTTTAC	[3]
HFR1-R TGAGGAGAAGAAGCTGGTGATG	[3]
IAA29-F ATAGCAAGAAAAGTGGATATCAAGC	This study
IAA29- R AAGTAGCCAGTCACCCTCTTTCCCT	This study
PIL1-F AAATTGCTCTCAGCCATTCGTGG	[4]
PIL1-R TTCTAAGTTTGAGGCGGACGCAG	[4]
PIF4-F ATGATGTCAATGGGATGTGGAATGAT	This study
PIF4-R GTGGTCCAAACGAGAACCGTCGGTGGTC	This study
PIF1-F TCCATGAAACAACTTACAACTCCATCA	[1]
PIF1-R CATCGTACTCACTCTTTGAAATCCACA	[1]
PIF3-F TCCATGAAACAACTTACAACTCCATCA	[1]
PIF3-R CATCGTACTCACTCTTTGAAATCCACA	[1]
PIF5-F TCTTCGCCTACGTTGACGGGAACGT	This study
PIF5-R CTCTAGCATTTTTGGCTGTCTAGGA	This study
phyB-F GTTGTCCTACTTGCCTTTCTAGTGG	This study
phyB-R TATTTTATTATTTTATTAAAAACCA	This study
phyB-F GTTGTCCTACTTGCCTTTCTAGTGG	This study
phyB-R TATTTTATTATTTTATTAAAAACCA	This study
myc-F GGGTTAATTAACGGTGAACAAAA	This study
myc-R GGAGATTAGCTTTTGTTCACCGT	This study
PIF1-R ATCGGTATCGAAGTCAGGGACAA (for TAP-PIF1)	This study
For ChIP-qPCR	
YUC8-ChIP-F GGGAATGGGTTTGATGTGGAATT	[2]
YUC8-ChIP-R GAGAAGGGAAGTGATGGAATTAG	[2]
IAA29-ChIP-F CTCACTACCTTTCGCTTAACGTG	[1]
IAA29-ChIP-R GTAGTATGTAAATCTAGACCCAA	[1]
HEC1-ChIP-F TGCCTTCTTTGCCTTTAGTG	[4]
HEC1-ChIP-R CCGTCGATAATTGACCAATG	[4]
HEC2-ChIP-F CCTTAGTTCACAACTCGTG	[4]
HEC2-ChIP-R TAGTCACTTGGTGTCACTAAA	[4]
PIF4-ChIP-F ATGTCCCAGAACTTGCCACG	[5]
PIF4-ChIP -R CAGGAGCATAAAGATATTACAGCGA	[5]

References:

1. Sun, J., et al., PIF4 and PIF5 Transcription Factors Link Blue Light and Auxin to Regulate the Phototropic Response in *Arabidopsis*. The Plant Cell, 2013. **25**(6): p. 2102-2114.
2. Sun, J., et al., PIF4–Mediated Activation of YUCCA8 Expression Integrates Temperature into the Auxin Pathway in Regulating *Arabidopsis* Hypocotyl Growth. PLOS Genetics, 2012. **8**(3): p. e1002594.
3. Xu, X., et al., Reciprocal proteasome-mediated degradation of PIFs and HFR1 underlies photomorphogenic development in *Arabidopsis*. Development, 2017. **144**(10): p. 1831-1840.
4. Zhu, L., R. Xin, and E. Huq, A Protein-Based Genetic Screening Uncovers Mutants Involved in Phytochrome Signaling in *Arabidopsis*. Frontiers in Plant Science, 2016. **7**(1086).
5. Zhai, H., et al., Cryptochrome 1 Inhibits Shoot Branching by Repressing the Self-Activated Transciption Loop of PIF4 in *Arabidopsis.* Plant Communications, 2020. **1**(3): p. 100042.
