# Supplementary figures and images for: An autoregulatory negative feedback loop controls thermomorphogenesis in Arabidopsis
Source: PLoS Genet. 2021 Jun 1;17(6):e1009595. doi: 10.1371/journal.pgen.1009595 (PMC8195427; doi:10.1371/journal.pgen.1009595)

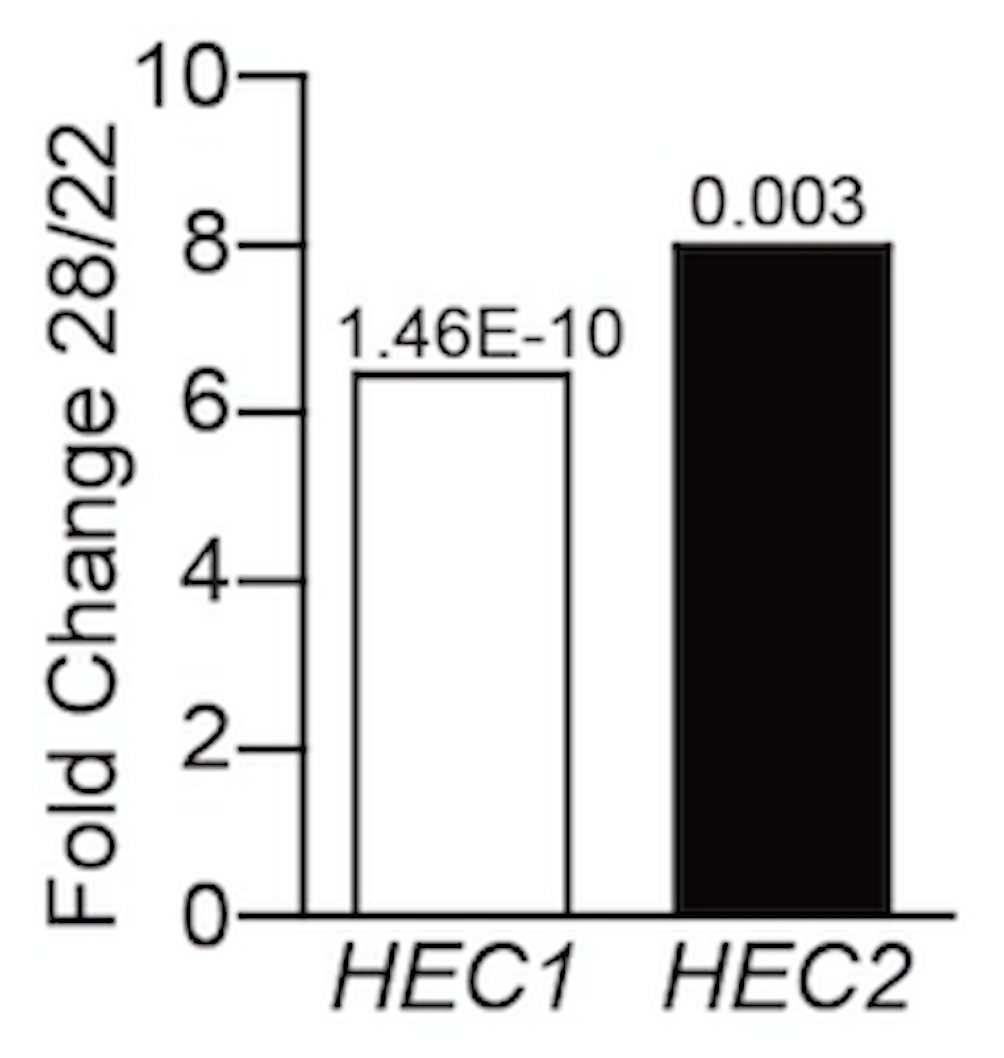

Supplement: S1 Fig — RNA-seq data show transcription level of HEC1 and HEC2 in WT comparing normal and high ambient temperature. Numbers in the bar graph indicate p-value. Sequence data were obtained from publicly available GEO web site under accession number GSE142354. (TIFF) [file pgen.1009595.s002.tiff]

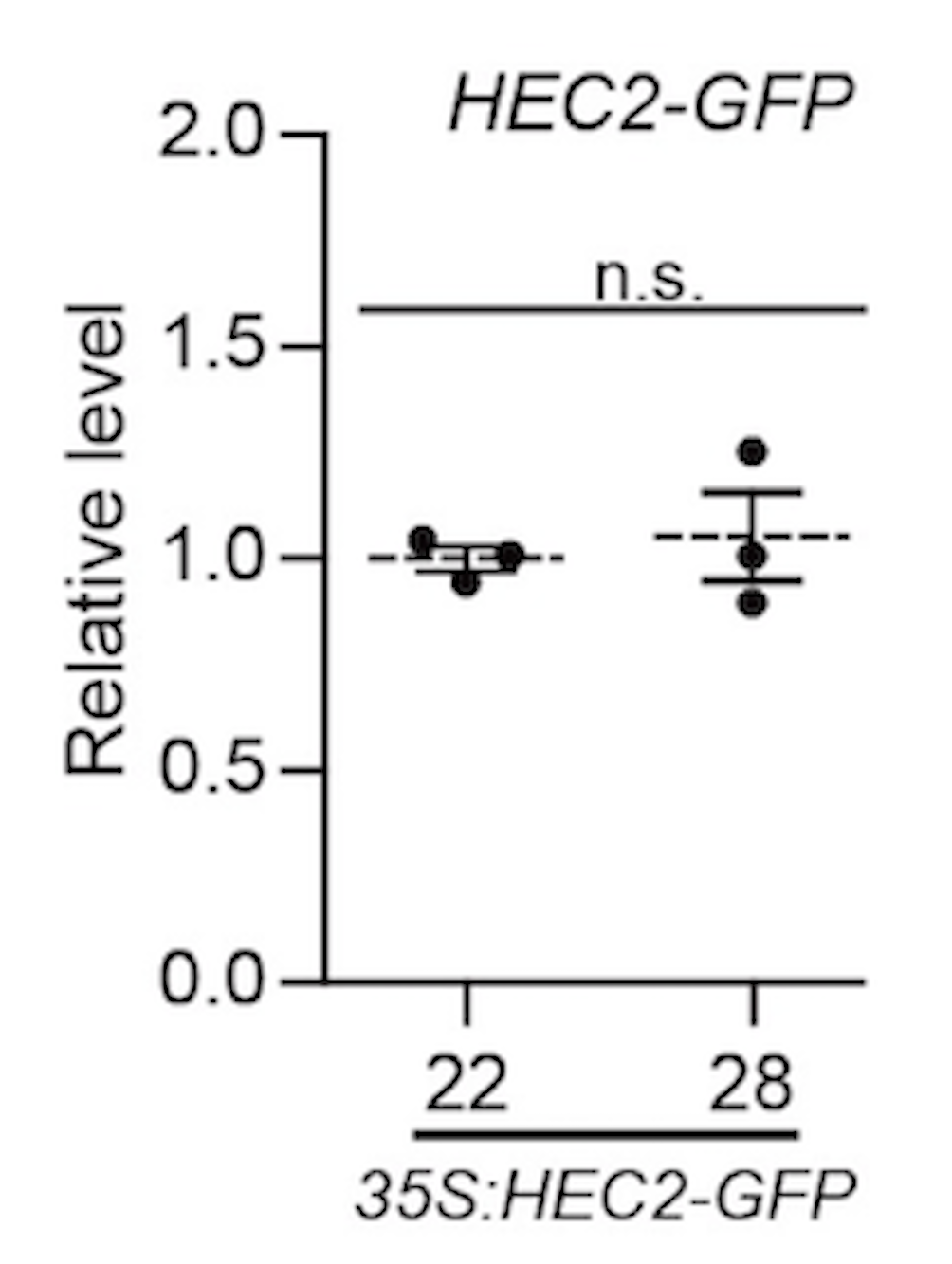

Supplement: S2 Fig — RT-qPCR was performed to detect HEC2-GFP transcript level. Samples were from 35S:HEC2-GFP whole seedling grown for 5 days in 22°C and transferred to 22°C or 28°C for 4 hours. Three biological replicates were used in this study. Relative gene expression levels were normalized using expression levels of ACT7. n.s. stands for not significant according to Student’s t-test (P<0.05). (TIFF) [file pgen.1009595.s003.tiff]

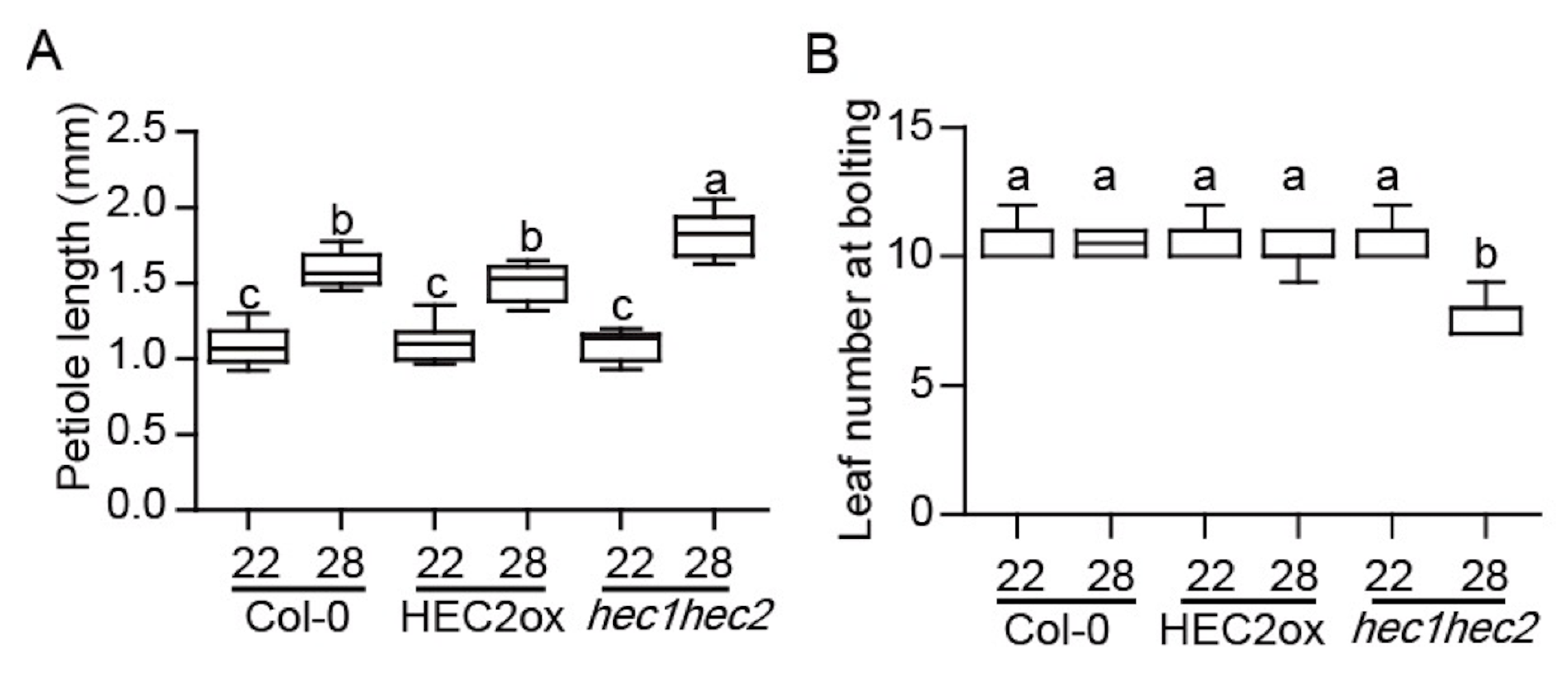

Supplement: S3 Fig — (A) Box plot shows the petiole lengths of genotypes indicated. Seedlings were grown for two days in continuous white light at 22°C and then either kept at 22°C or transferred to 28°C for additional 5 days. More than 10 seedlings were measured. The letters a-c indicate statistically significant differences based on one-way ANOVA analysis with Tukey’s HSD test. Tukey’s box plot was used with median as a center value. (B) Box plot shows the leaf number for bolting under long day conditions (16L:8D). Seedlings were grown for two days in continuous white light at 22°C and then either kept at 22°C or transferred to 28°C until bolting. More than 10 seedlings were measured. The letters a-b indicate statistically significant differences based on one-way ANOVA analysis with Tukey’s HSD test. Tukey’s box plot was used with median as a center value. (TIFF) [file pgen.1009595.s004.tiff]

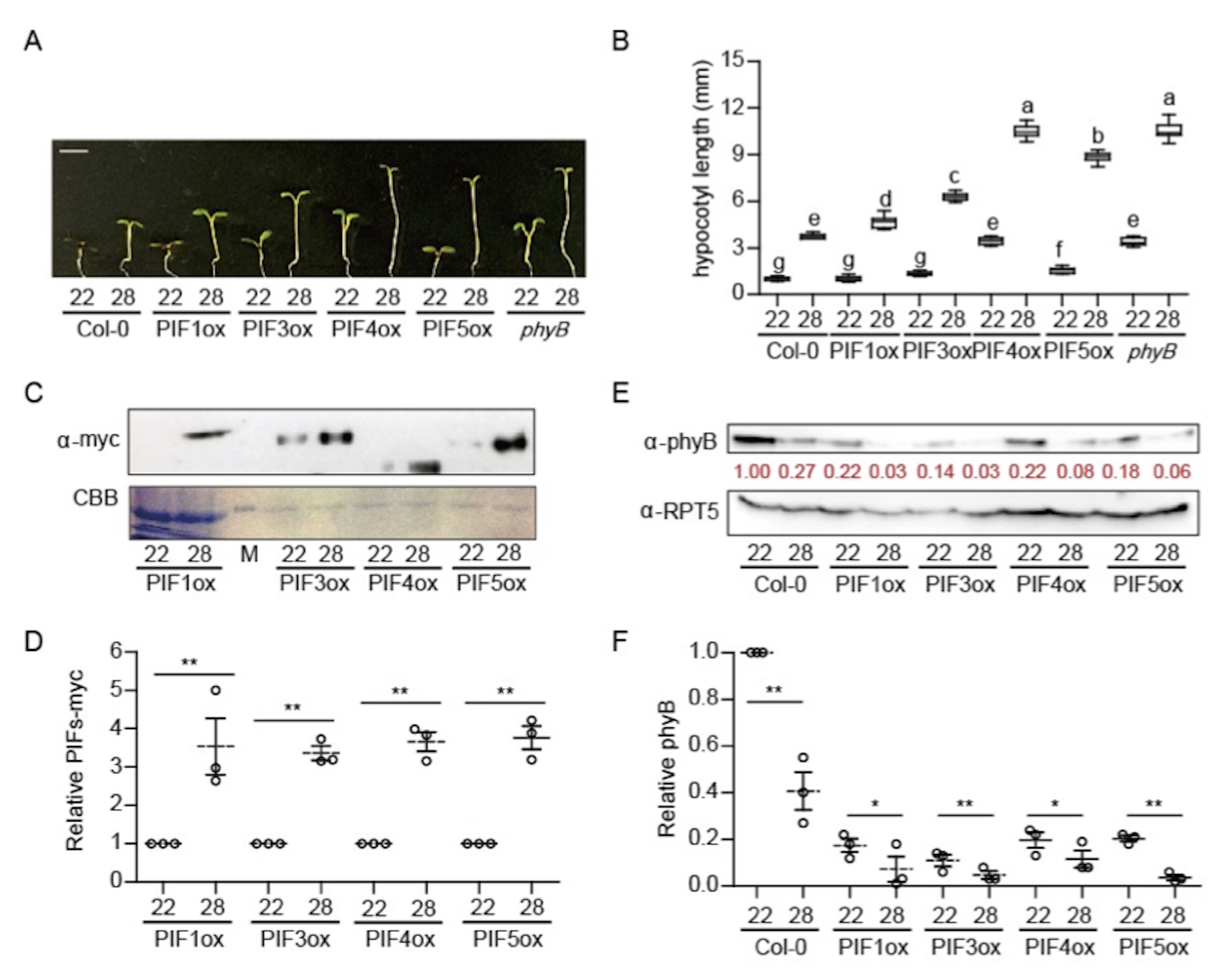

Supplement: S4 Fig — (A) Photograph shows seedling phenotypes of PIFs-overexpression lines and phyB-9 at normal and high ambient temperature. Seedlings were grown for two days in continuous white light at 22°C and then either kept at 22°C or transferred to 28°C for additional four days before being photographed. The scale bar represents 5 mm. (B) Box plot shows the hypocotyl lengths of seedlings described in (A). More than 10 seedlings were measured for each experiment and was repeated 3 times. The letters a-c indicate statistically significant differences between means of hypocotyl lengths (P<0.05) based on one-way ANOVA analysis with Tukey’s HSD test. Tukey’s box plot was used with median as a center value. (C, E) Western blots show the level of PIFs (C) and phyB (E) in Wild-type and various PIF overexpression lines. Seedlings were grown for 5 days at 22°C and either kept at 22°C or transferred to 28°C for 4 hours. Coomassie staining or anti-RPT5 was used as a control. Red number indicates the quantitation value from anti-phyB or anti-myc detection divided by the control. (D and F) Dot plots show the relative amount of PIF4 (D, n = 3) or phyB (F, n = 3). Asterisks indicate statistically significant difference using Student’s t-test; *p < 0.05 and **p < 0.01. (TIFF) [file pgen.1009595.s005.tiff]

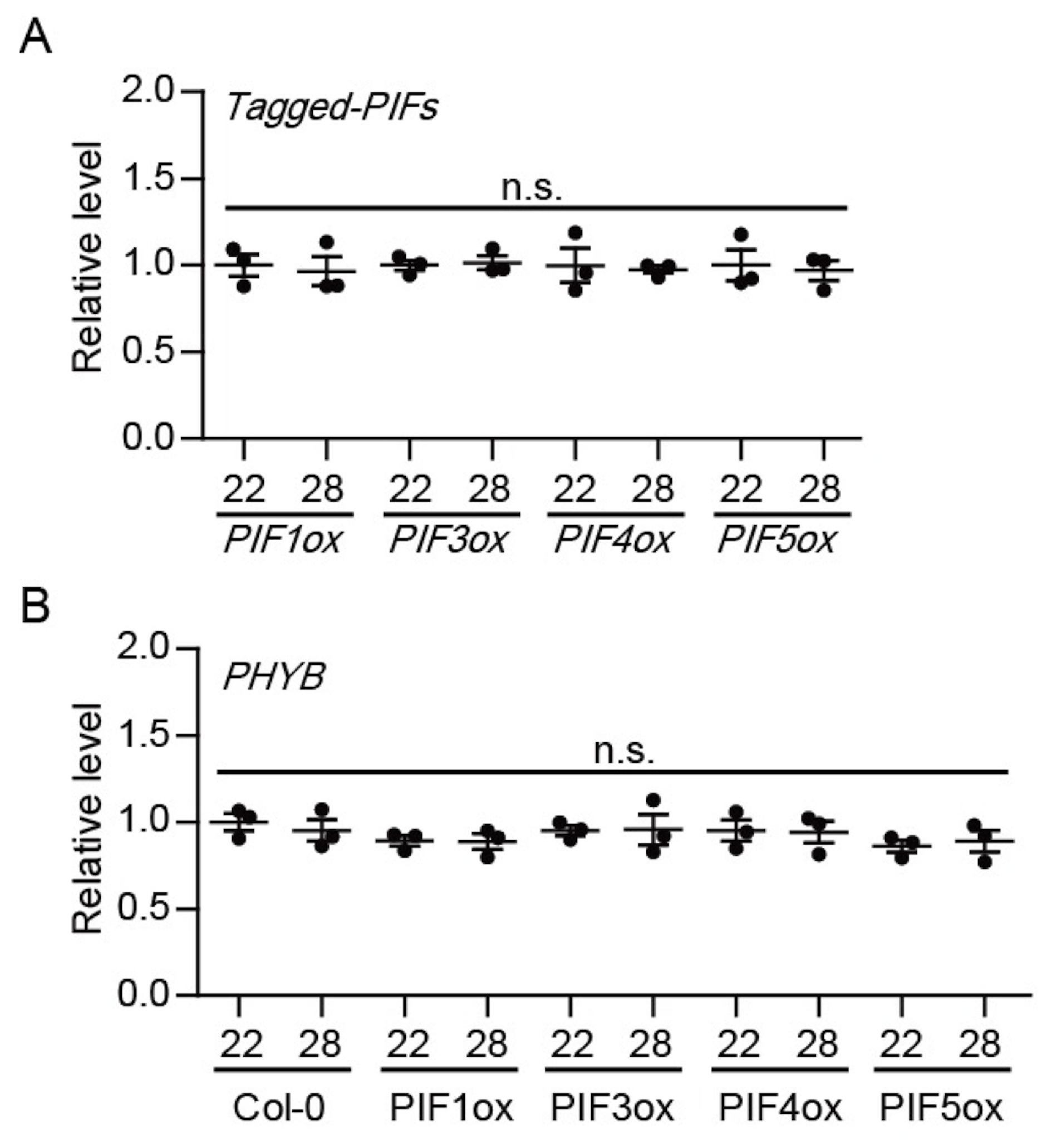

Supplement: S5 Fig — RT-qPCR was performed to detect tagged-PIFs and PHYB transcript levels. Samples were from WT, 35S:TAP-PIF1, PIF3-myc, PIF4-myc and PIF5-myc seedlings grown for 5 days at 22°C and transferred to 22°C or 28°C for 4 hours. Three biological replicates were used in this study. Relative gene expression levels were normalized using the expression level of ACT7. n.s. stands for not significant according to Student’s t-test (P<0.05). (TIFF) [file pgen.1009595.s006.tiff]

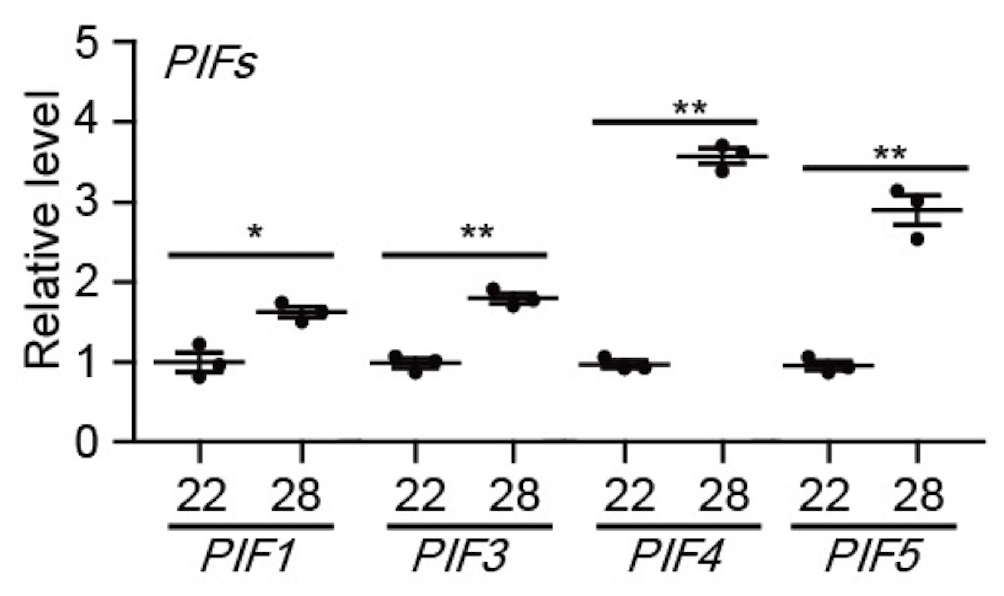

Supplement: S6 Fig — RT-qPCR was performed to detect the transcript levels of four major PIFs (PIF1, PIF3, PIF4 and PIF5). Samples were from WT seedling grown for 5 days at 22°C and transferred to 22°C or 28°C for 4 hours. Three biological replicates were used in this study. Relative gene expression levels were normalized using the expression level of ACT7. Asterisks indicate statistically significant difference using Student’s t-test; *p < 0.05 and **p < 0.01. (TIFF) [file pgen.1009595.s007.tiff]

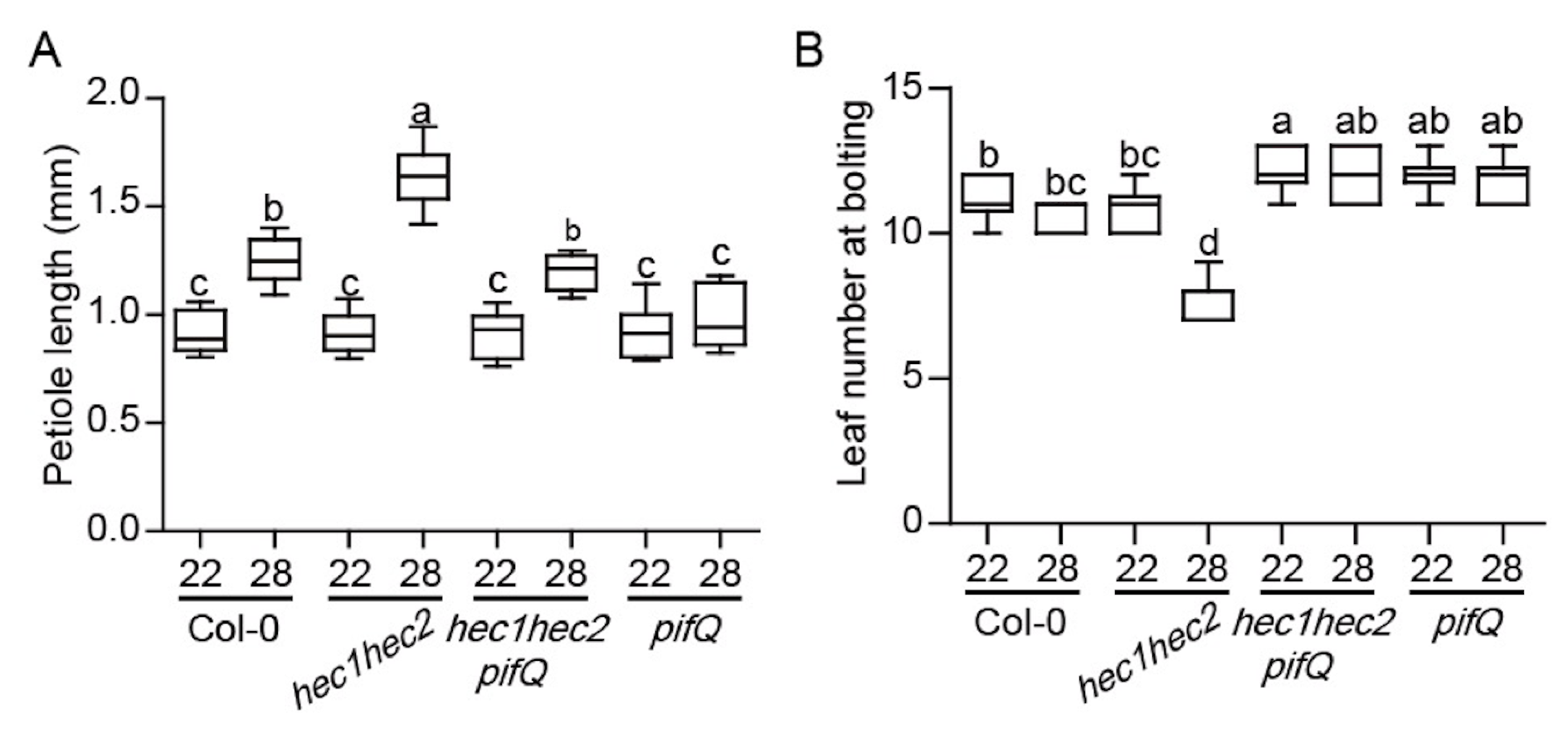

Supplement: S7 Fig — (A) Box plot shows the petiole lengths of genotypes indicated. Seedlings were grown for two days in continuous white light at 22°C and then either kept at 22°C or transferred to 28°C for additional 5 days. More than 10 seedlings were measured. The letters a-c indicate statistically significant differences based on one-way ANOVA analysis with Tukey’s HSD test. Tukey’s box plot was used with median as a center value. (B) Box plot shows the leaf number for bolting under long day conditions (16L:8D). Seedlings were grown for two days in continuous white light at 22°C and then either kept at 22°C or transferred to 28°C until bolting. More than 10 seedlings were measured. The letters a-b indicate statistically significant differences based on one-way ANOVA analysis with Tukey’s HSD test. Tukey’s box plot was used with median as a center value. (TIFF) [file pgen.1009595.s008.tiff]

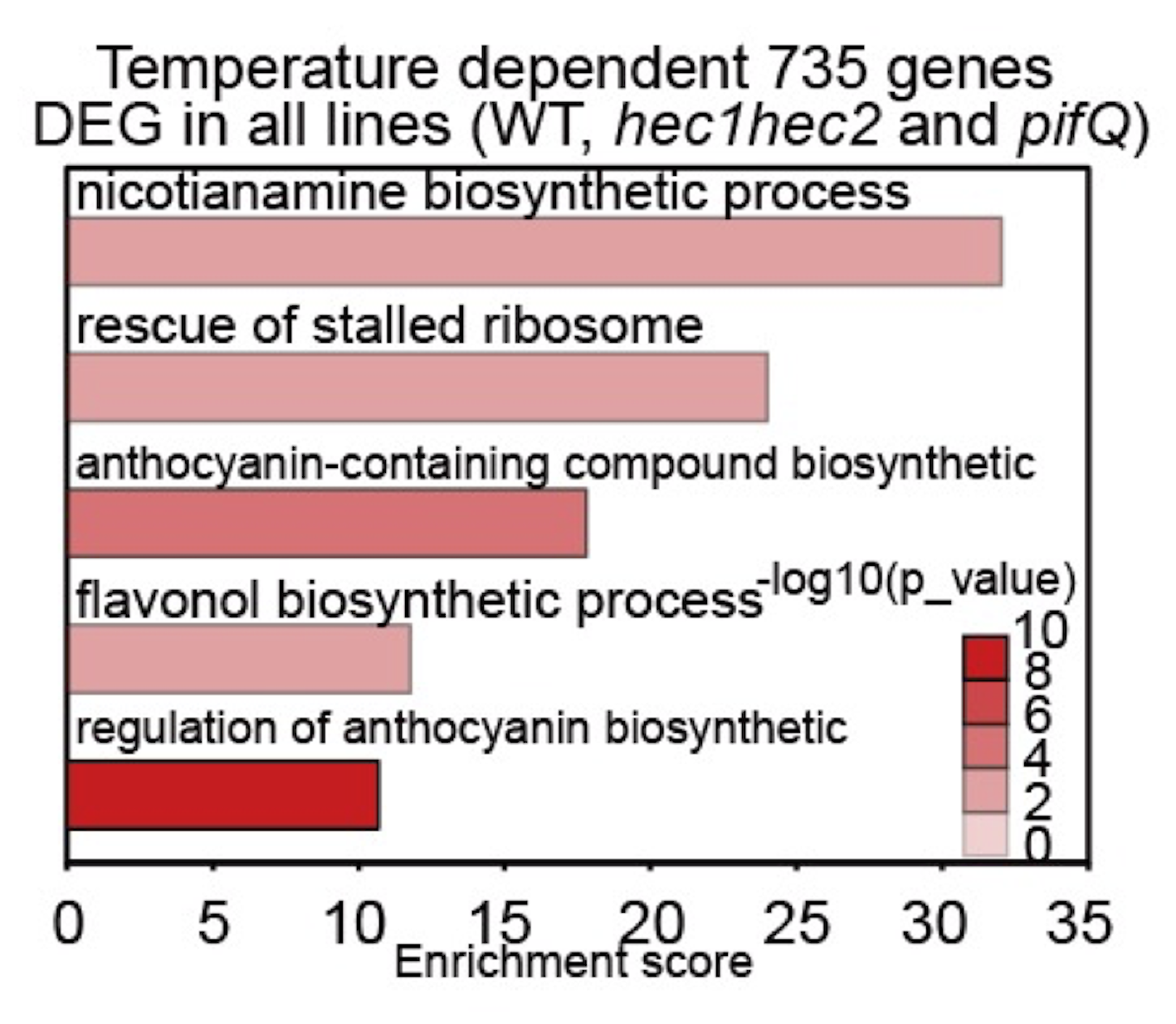

Supplement: S8 Fig — Gene Ontology (GO) analysis of temperature-dependent 735 genes that are common among three genotypes. Six-day-old white light-grown seedlings were transferred to 22°C or 28°C for additional 24 hours and total RNA was extracted from three biological replicates for RNA-seq analyses. (TIFF) [file pgen.1009595.s009.tiff]

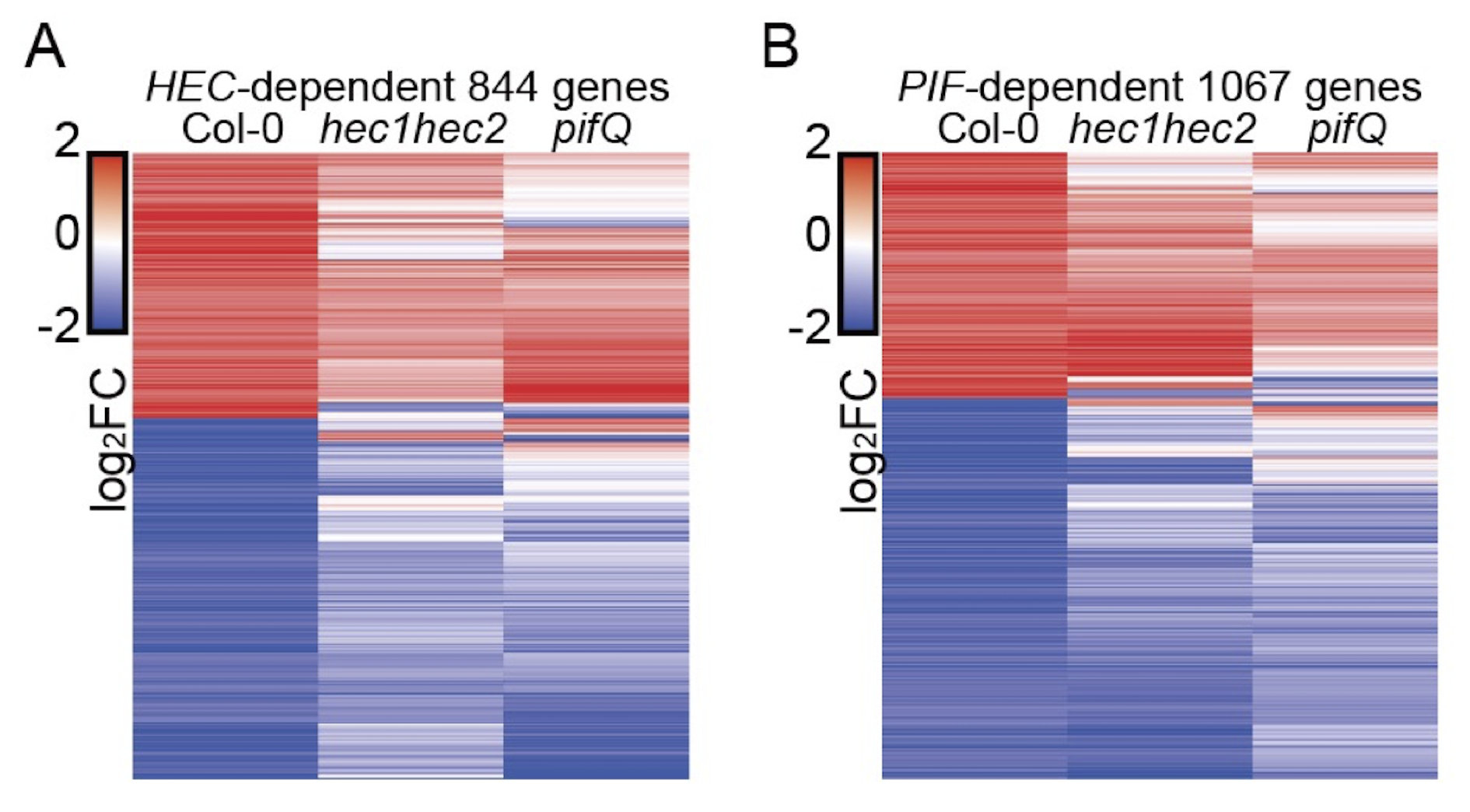

Supplement: S9 Fig — (A-B) Hierarchical clustering displaying 844 (638+206) HEC-dependent DEGs (A) and 1067 (638+429) PIF-dependent DEGs (B) shows distinct pattern in hec1hec2 and pifQ mutant at high ambient temperature. (TIFF) [file pgen.1009595.s010.tiff]

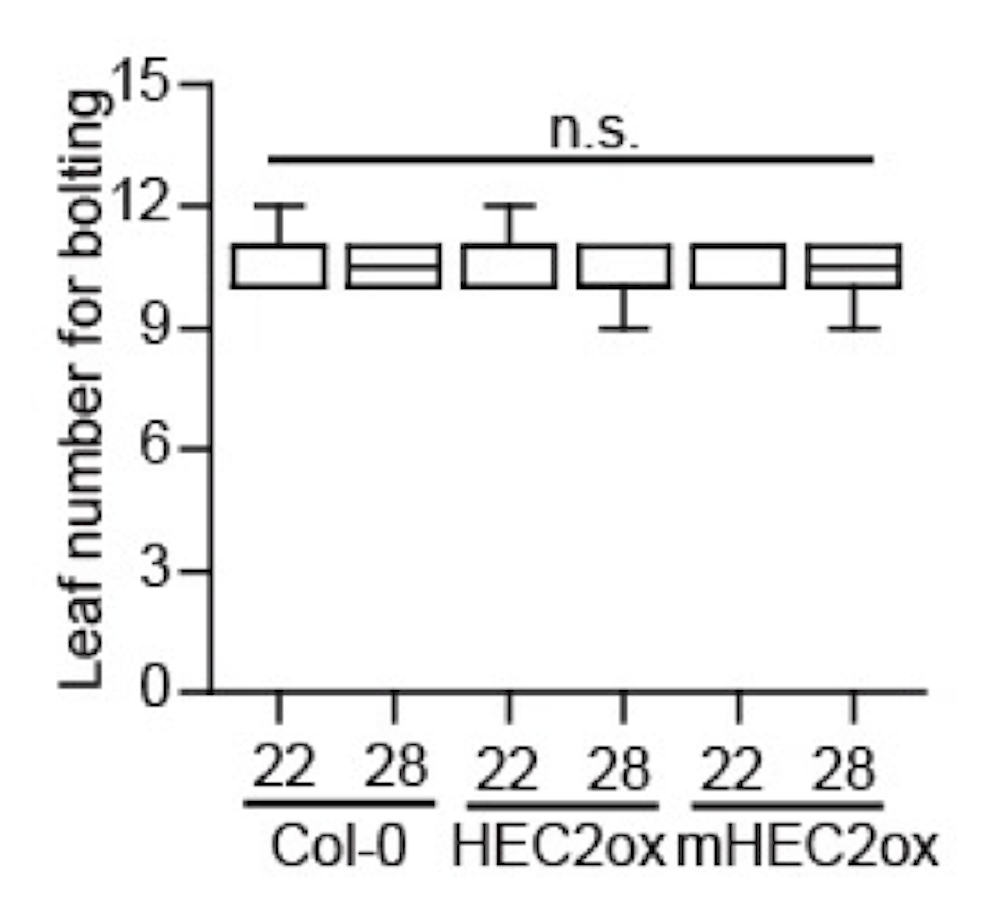

Supplement: S10 Fig — (A) Box plot shows the leaf number for bolting (16L:8D). More than 10 seedlings were measured. n.s. stands for not significant based on one-way ANOVA analysis with Tukey’s HSD test. Tukey’s box plot was used with median as a center value. (TIFF) [file pgen.1009595.s011.tiff]

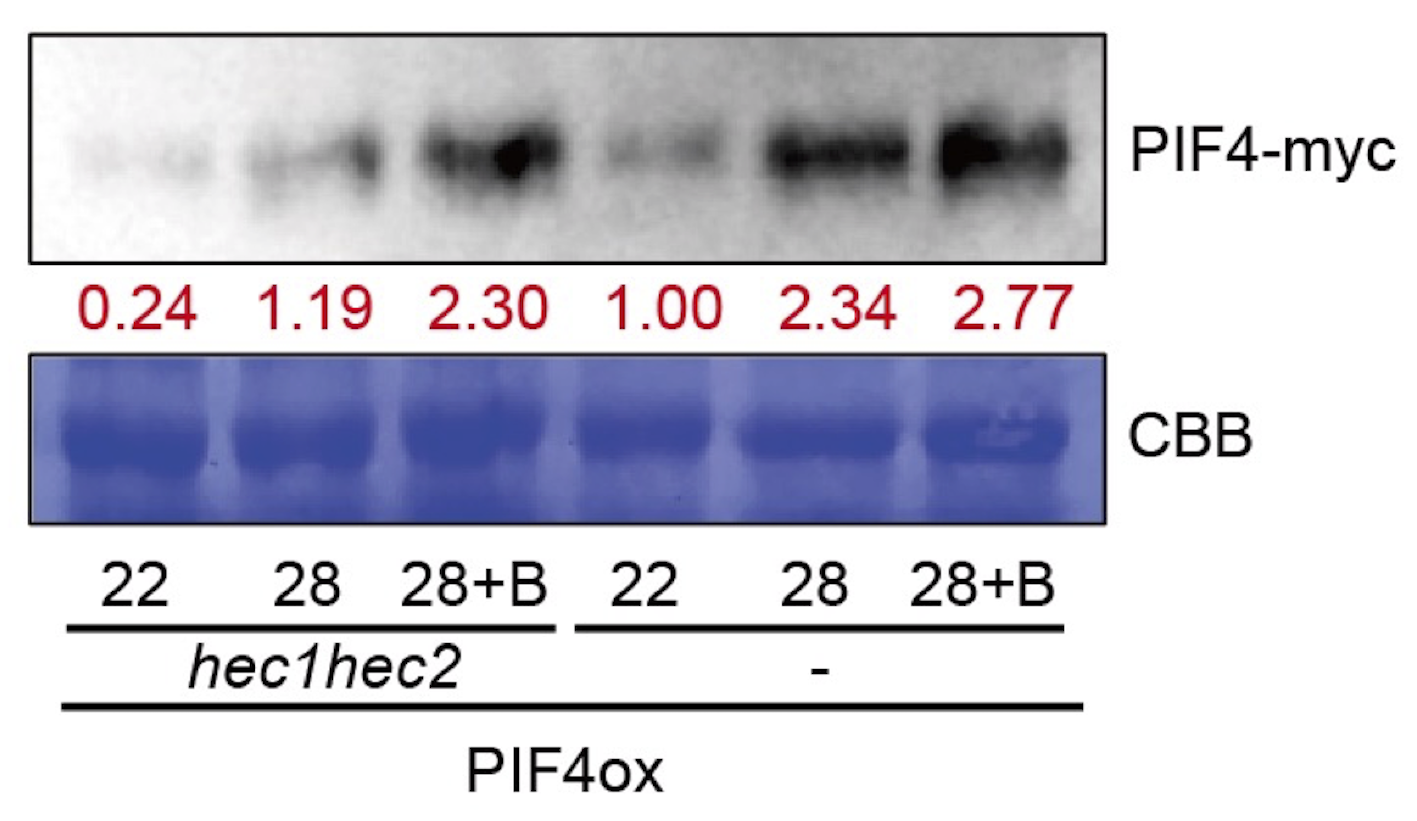

Supplement: S11 Fig — (A) Western blot shows the level of PIF4-myc from whole seedlings of 35S:PIF4-myc in either hec1hec2 or wild-type backgrounds. Seedlings were grown for 5 days at 22°C and either kept at 22°C or transferred to 28°C for 4 hours. B stands for bortezomib treatment. Coomassie staining was used as a control. Red number indicates the quantitation value from anti-myc divided by the control. (TIFF) [file pgen.1009595.s012.tiff]

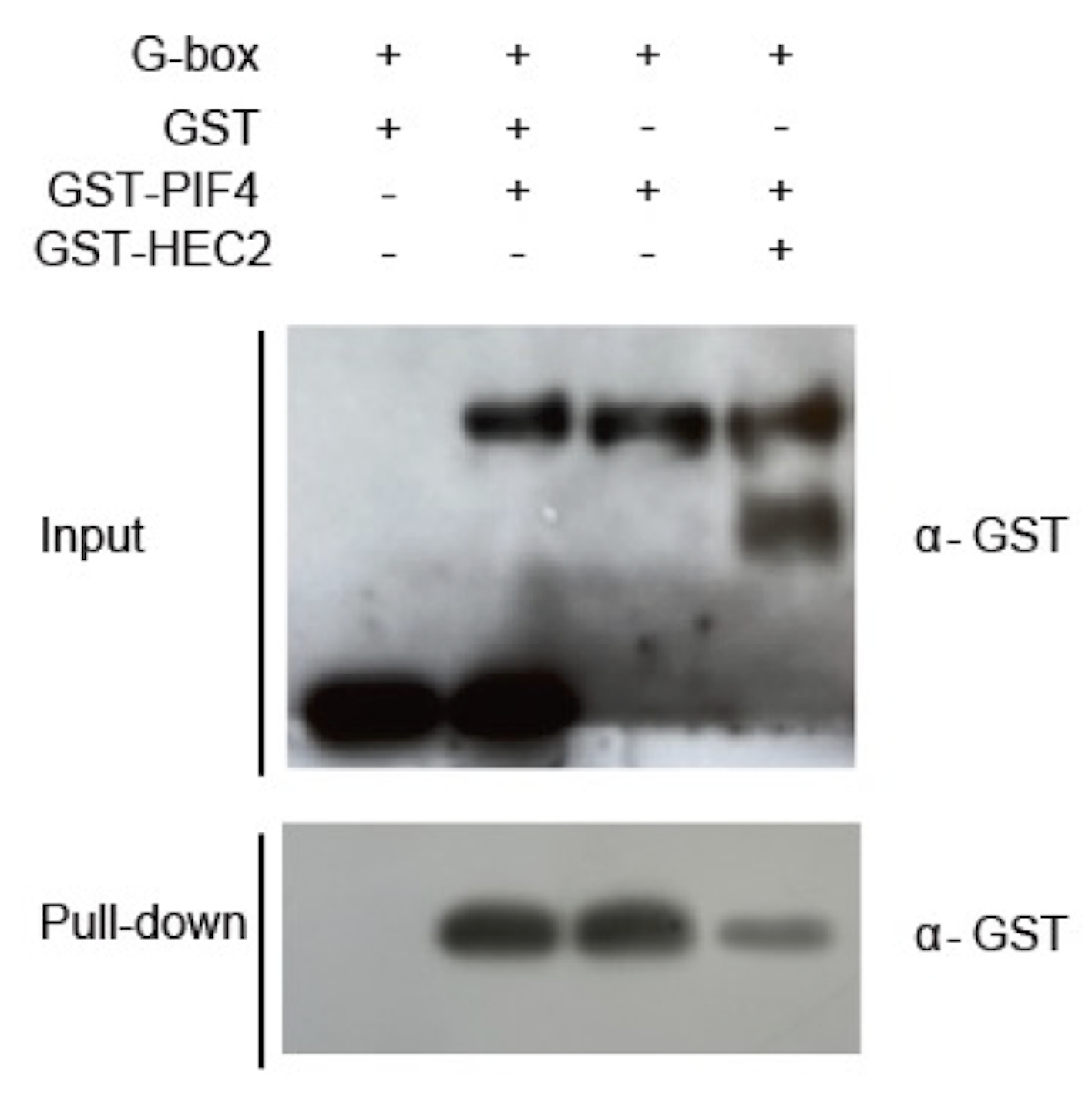

Supplement: S12 Fig — (Upper panel) Immunoblot shows the amount of GST only (as a control), GST-PIF4, and GST-HEC2 and their combinations as input. All GST-fusion proteins were expressed and purified from E. coli and detected using anti-GST antibody. (Lower panel) Immunoblot shows the amount of GST-PIF4 bound to the DNA. Biotin labeled PIF4 G-box promoter region was precipitated using streptavidin beads after pre-binding with combinations of proteins as indicated by + and/or -. (TIFF) [file pgen.1009595.s013.tiff]

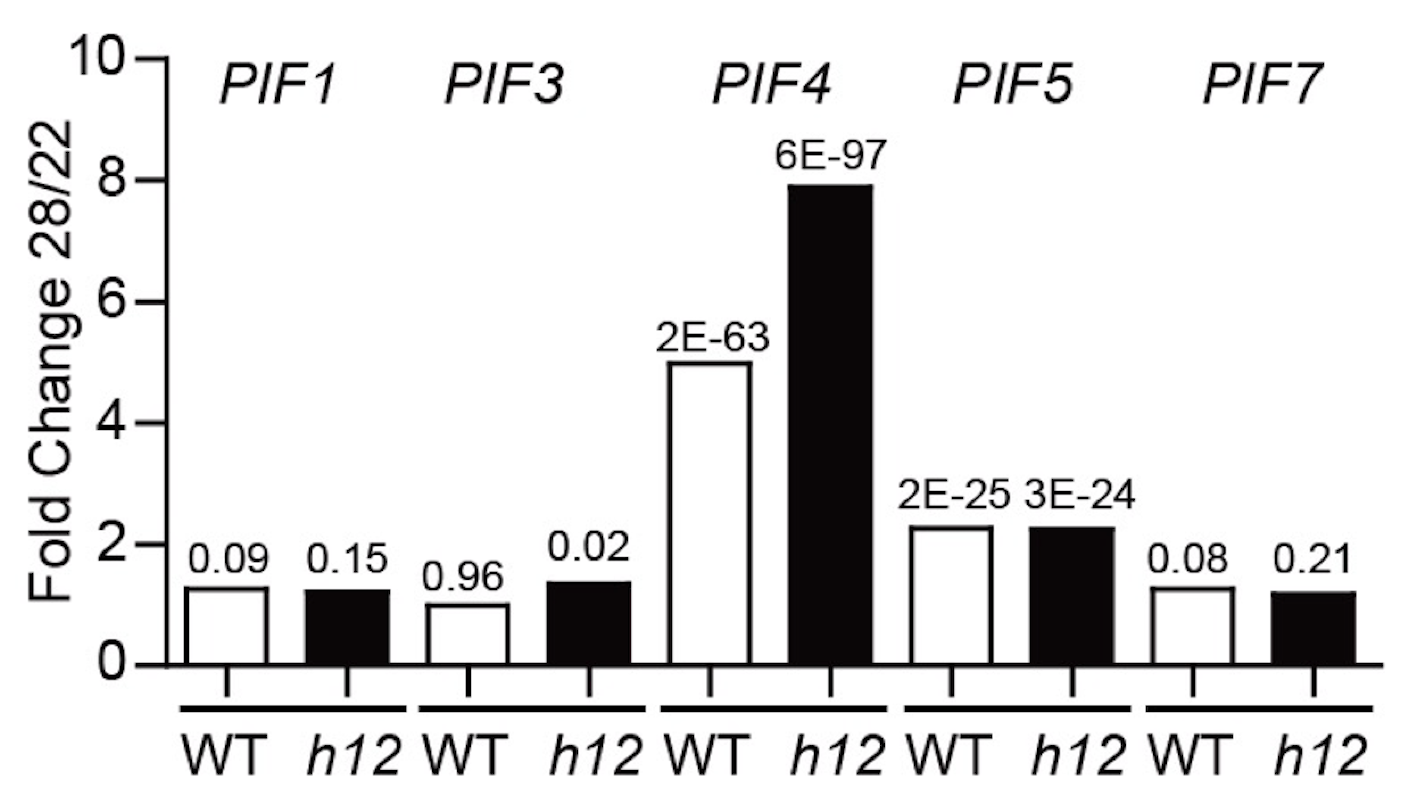

Supplement: S13 Fig — RNA-seq data show transcription level of PIF1, PIF3, PIF4, PIF5, and PIF7 in WT and hec1hec2 background comparing normal and high ambient temperature. h12 indicates hec1 hec2 mutant. Numbers in the bar graph indicate p-value. (TIFF) [file pgen.1009595.s014.tiff]
